# Surveillance of mother to child transmission of HIV in Romania, a 12 years’ experience in the National Institute for Infectious Diseases “Prof. Dr. Matei Balş”

**DOI:** 10.1186/1471-2334-13-S1-O1

**Published:** 2013-12-16

**Authors:** Mariana Mărdărescu, Cristina Petre, Adrian Streinu-Cercel, Sorin Petrea, Ruxandra Neagu-Drăghicenoiu, Rodica Ungurianu, Ana Maria Tudor, Alina Cibea, Delia Vlad, Mihai Mitran, Otilia Benea, Dan Oțelea, Carmen Crăciun, Tatiana Colțan, Marieta Iancu, Ionel Ionel, Alexandra Mărdărescu

**Affiliations:** 1 National Institute for Infectious Diseases "Prof. Dr. Matei Balş", Bucharest, Romania; 2 Carol Davila University of Medicine and Pharmacy, Bucharest, Romania; 3 Clinical Hospital of Obstetrics and Gynecology “Prof. Dr. Panait Sârbu”, Bucharest, Romania; 4 National Centre for Transmissible Diseases Surveillance and Control (CNSCBT), Bucharest, Romania; 5 Romanian HIV/AIDS Centre, National Institute for Infectious Diseases "Prof. Dr. Matei Balş", Bucharest, Romania

## Background

The latest WHO data regarding HIV/AIDS infection have revealed the real dimension of this epidemic. In Romania, in 2012, out of the 754 new detected cases, 19 represented children between 0-14 years. Vertical transmission was recorded for 16 (2.6%) of these cases. Within this epidemiological context, mother to child transmission continues to represent a top theme, which determined us to continue with the observations initiated in 2000 on this phenomenon.

## Methods

During 01 January 2000 – 31 December 2012, children from all around the country were included in the study and assessed at the National Institute for Infectious Diseases “Prof. Dr. Matei Balş” in Bucharest. They were surveyed clinically and biologically until the age of 18 months. Relevant data on the children were recorded: gender, age, time of diagnosis, antiretroviral therapy (ART) prophylaxis (YES or NO), type of birth and nourishment, CD4 count, viral load (VL) at baseline and at the end of the surveillance period. When assessing the mothers we focused on: age, environment, level of education, occupation, time of HIV infection diagnosis, treatment/prophylaxis, type of birth, CD4 and VL at birth (data obtained from medical records or anamnesis).

## Results

We surveyed 517 children with ages between 0-18 months. Of these children under observation for 12 years, 15% were considered infected with HIV. This rate decreased in stages, from 45% in 2005 to 17% in 2010. The main transmission causes were late HIV infection diagnosis of the mother, lack of prophylaxis/treatment for mothers, vaginal birth, breastfeeding, lack of prophylaxis in children or tardiness in initiating it.

## Conclusion

Despite active measures, the rate of HIV transmission remains very high. The elimination of vertical transmission is considered a realistic objective of public health policies. The collaboration and active involvement of specialists from various medical and social domains is a necessity.

